# A Missense Mutation in the *SERPINH1* Gene in Dachshunds with Osteogenesis Imperfecta

**DOI:** 10.1371/journal.pgen.1000579

**Published:** 2009-07-24

**Authors:** Cord Drögemüller, Doreen Becker, Adrian Brunner, Bianca Haase, Patrick Kircher, Frank Seeliger, Michael Fehr, Ulrich Baumann, Kerstin Lindblad-Toh, Tosso Leeb

**Affiliations:** 1 Institute of Genetics, Vetsuisse Faculty, University of Berne, Berne, Switzerland; 2 Department of Clinical Veterinary Medicine, Vetsuisse Faculty, University of Berne, Berne, Switzerland; 3 Astra Zeneca Safety Assessment, R&D Södertälje, Södertälje, Sweden; 4 Clinic for Small Animals, University of Veterinary Medicine Hannover, Hannover, Germany; 5 Department of Chemistry and Biochemistry, University of Berne, Berne, Switzerland; 6 Broad Institute of Harvard and MIT, Cambridge, Massachusetts, United States of America; 7 Department of Medical Biochemistry and Microbiology, Uppsala University, Uppsala, Sweden; Stanford University School of Medicine, United States of America

## Abstract

Osteogenesis imperfecta (OI) is a hereditary disease occurring in humans and dogs. It is characterized by extremely fragile bones and teeth. Most human and some canine OI cases are caused by mutations in the *COL1A1* and *COL1A2* genes encoding the subunits of collagen I. Recently, mutations in the *CRTAP* and *LEPRE1* genes were found to cause some rare forms of human OI. Many OI cases exist where the causative mutation has not yet been found. We investigated Dachshunds with an autosomal recessive form of OI. Genotyping only five affected dogs on the 50 k canine SNP chip allowed us to localize the causative mutation to a 5.82 Mb interval on chromosome 21 by homozygosity mapping. Haplotype analysis of five additional carriers narrowed the interval further down to 4.74 Mb. The *SERPINH1* gene is located within this interval and encodes an essential chaperone involved in the correct folding of the collagen triple helix. Therefore, we considered *SERPINH1* a positional and functional candidate gene and performed mutation analysis in affected and control Dachshunds. A missense mutation (c.977C>T, p.L326P) located in an evolutionary conserved domain was perfectly associated with the OI phenotype. We thus have identified a candidate causative mutation for OI in Dachshunds and identified a fifth OI gene.

## Author Summary

Osteogenesis imperfecta (OI) is a genetic condition of humans and dogs characterized by extremely fragile bones and teeth. Most human OI cases are caused by defects in one of two collagen genes. Mutations in two other genes related to collagen maturation can also lead to OI in some patients. We studied Dachshunds with OI and initially investigated the two known collagen genes that are normally mutated in OI but did not find a mutation. Subsequently, we performed a search for shared segments across the entire genome in five affected Dachshunds. This experiment revealed that the causative mutation for OI in Dachshunds is located on dog chromosome 21. The *SERPINH1* gene known to be involved in collagen maturation is located in this shared genome region. We sequenced the *SERPINH1* gene in healthy and affected Dachshunds and found a single mutation exclusively shared by all affected dogs but not by healthy controls. Thus we have identified *SERPINH1* as a fifth OI gene and a mutation within this gene as the most likely cause of OI in Dachshunds. The knowledge of this mutation enables genetic testing and will allow breeders to eradicate the deleterious allele from the Dachshund breeding population. *SERPINH1* mutations might also be responsible for some human OI forms, where the causative mutation has not yet been identified.

## Introduction

Collagen I is the most abundant protein in the human body and its highly ordered fibril structure is responsible for its special mechanical properties. Together with inorganic hydroxylapatite it is the main component of bones and gives them elasticity while the hydroxylapatite alone would be very brittle. Defects in the structure of the highly ordered collagen I triple helix lead to osteogenesis imperfecta (OI), a disease characterized by extremely fragile bones and teeth. OI is sometimes also accompanied by blue sclera, hearing loss, dwarfism, dentinogenesis imperfecta, and other complications. Seven subtypes of human OI are distinguished based on the underlying genetic defects and phenotypic severity [1]. OI affects an estimated 6 to 7 per 100,000 people worldwide [http://ghr.nlm.nih.gov/condition=osteogenesisimperfecta/]. Approximately 85–90% of the human OI cases are caused by mutations in the *COL1A1* or *COL1A2* genes encoding the two different subunits of collagen I. More than 800 distinct mutations in these two genes have been described and most of them lead to autosomal dominant forms of OI [2]. The maturation and correct folding of collagens is a complicated process, which involves a large number of accessory proteins and chaperones. Recently mutations in two of these accessory proteins were found in patients with autosomal recessive forms of OI [3]–[7]. Both of these proteins are involved in the 3-hydroxylation of a specific proline residue in collagen I. One represents the enzymatically active prolyl-3-hydroxylase 1 itself and is encoded by the *LEPRE1* gene [3]. The other is called cartilage-associated protein (CRTAP) and forms a complex with the prolyl-3-hydroxylase [4]. For some human OI cases the underlying mutation has not yet been found.

OI also occurs in dogs and the dog may represent a better model for human OI than genetically engineered mice because of its larger body size and the resulting similarity of mechanical forces that act on the skeleton. OI in dogs has been described in Golden Retrievers, Beagles, Collies, Poodles, Norwegian Elkhounds, and Bedlington Terriers [8]–[12]. In Golden Retrievers a *COL1A1* mutation and in Beagles a *COL1A2* mutation has been reported to cause OI [8],[9]. For other canine OI cases the underlying genetic defect has not been elucidated. We have observed a severe form of OI in rough-coated Dachshunds that is inherited as a monogenic autosomal recessive trait [13]. In our initial analysis of the OI Dachshunds we did not find any mutations in the *COL1A1* or *COL1A2* genes. Therefore, we hypothesized that a mutation in a novel OI gene may be responsible for the observed bone defects in Dachshunds. Consequently, we started a positional cloning approach to identify this mutation.

## Results

### Collection of informative families and exclusion of *COL1A1* and *COL1A2*

**Figure 1:**
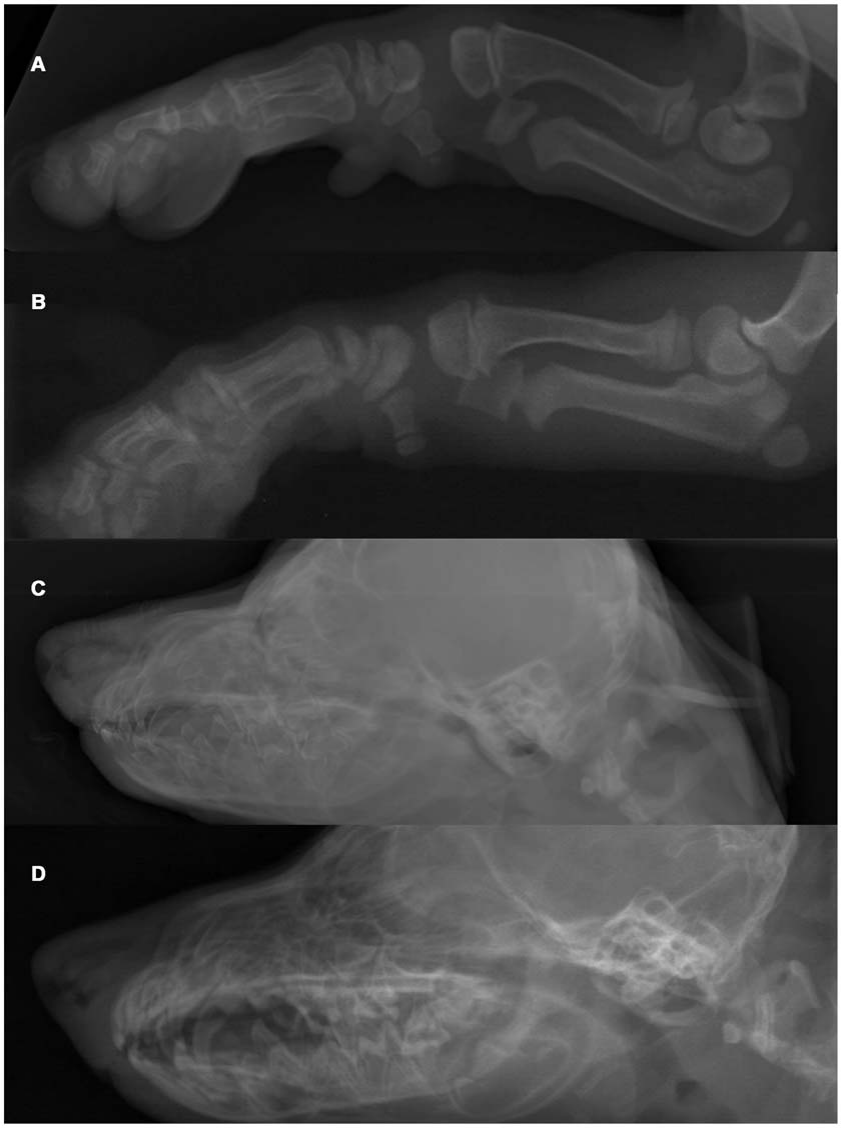
Radiographs of an OI affected and a control Dachshund demonstrating generalized osteopenia in canine OI. (A) Foreleg of an affected Dachshund. Note the overall decreased radiopacity of the skeleton with the thin compact bone and inhomogeneous, shallow trabeculation in the entire foreleg. No pathologic fractures were seen in this puppy. (B) Foreleg of a control Dachshund. (C) Skull of an affected puppy. There is decreased opacity and poor delineation of the skull. Note the lack of visualization of the lamina dura of the dental alveoli leading to a “floating” appearance of the teeth, which themselves show a lack of mineralization. (D) Skull of a control dog.

We collected samples from six Dachshund families segregating for congenital OI (Figure 1; Video S1). The parents of all available cases were healthy (Figure S1). The pedigrees were consistent with a monogenic autosomal recessive inheritance although the ratio of affected Dachshunds from the presumed carrier x carrier matings was slightly higher than expected with 14 out of 36 total pups affected instead of the expected 9/36. The available pedigree records indicate that the affected dogs from the German Dachshund breeding population share common ancestors and most likely trace back to a single common founder. As the OI phenotype in Dachshunds shows striking clinical similarities to human OI forms, we initially hypothesized that mutations in *COL1A1* or *COL1A2* might cause the canine disease. In order to validate whether a mutation in one of these genes might be responsible for OI, we genotyped three gene associated microsatellite markers derived from the surrounding genome sequence of *COL1A1* (located on chromosome 9 (CFA 9) at 29.5 Mb) and *COL1A2* (located on CFA 14 at 22.8 Mb), respectively. Two-point linkage analysis in the six available families clearly excluded the *COL1A2* gene but indicated a suggestive linkage of OI to the region of *COL1A1* with a positive LOD score of 1.5 (Table S1). However, the re-sequencing of *COL1A1* using DNA of four affected and four healthy Dachshunds did not reveal any disease associated sequence polymorphism within the 51 coding exons and flanking intron regions of the *COL1A1* gene. Furthermore, haplotype analysis revealed five different CFA 9 microsatellite marker haplotypes in affected dogs, which was not compatible with our assumption of a single founder mutation in all OI affected dogs.

### Mapping of the causative mutation

**Figure 2:**
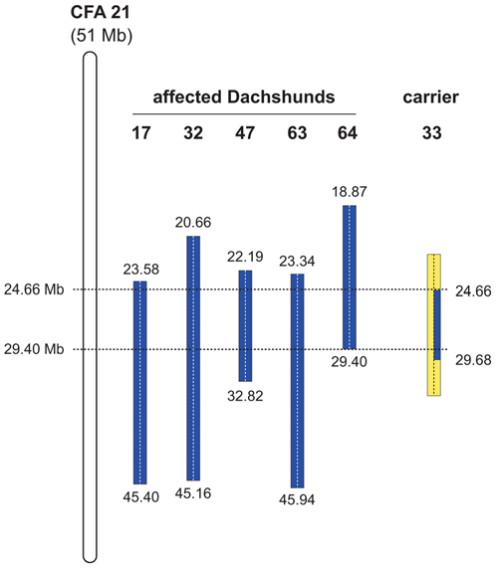
Mapping of the OI mutation. SNP genotypes of selected CFA 21 markers are shown. Alternate SNP alleles are represented in blue and yellow. The two copies of CFA 21 for each dog are separated by a vertical dashed line. The analysis of SNP genotypes from five affected dogs indicated that they had extended homozygous regions on CFA 21 (indicated as blue blocks). The boundaries of these homozygous blocks are given in Mb. All five affected dogs had homozygous intervals with shared alleles between 23.58 Mb and 29.40 Mb. We also genotyped five parents of affected dogs assumed to be carriers of the mutation. One of these carriers, animal no. 33, had one copy of the disease-associated haplotype in the critical interval (indicated in blue). In comparison to the affected dogs it was homozygous for the opposite SNP alleles (indicated in yellow) at several positions proximal of 24.66 Mb and distal of 29.40 Mb. Thus – assuming that it resides on the common blue haplotype block – the causative mutation is located within the interval from 24.66 Mb to 29.40 Mb on CFA 21.

Based on the pedigrees of our samples we hypothesized that the affected Dachshunds most likely were inbred to one single founder animal. Under this scenario the affected Dachshunds were expected to be identical by descent (IBD) for the causative mutation and flanking chromosomal segments. Therefore, we decided to apply a homozygosity mapping approach to determine the position of the mutation in the canine genome. We genotyped approximately 50,000 evenly spaced SNPs from five affected dogs and five obligate carriers. We analyzed the cases for extended regions of homozygosity with simultaneous allele sharing. Only one genome region fulfilled our search criteria (Table S2). On CFA 21 all five affected genotyped dogs were homozygous and shared identical alleles over 102 SNP markers corresponding to a 5.82 Mb interval from 23.58–29.40 Mb (Figure 2). We then examined the five obligate carriers for the mutation and reconstructed one copy of the disease-associated haplotype in each dog. One of the carriers showed a recombination event, which allowed us to narrow down the critical interval harboring the causative mutation to 4.74 Mb from 24.66–29.40 Mb (Figure 2).

### Identification of a functional candidate gene and mutation analysis

**Figure 3:**
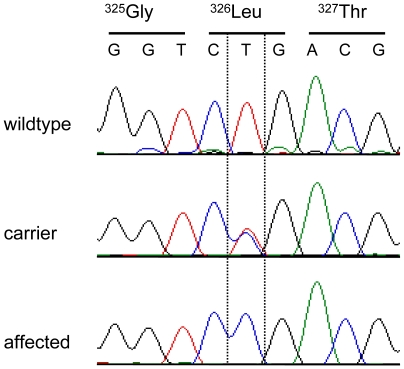
Electropherograms of the *SERPINH1* c.977T>C mutation. Representative sequence traces of PCR products amplified from genomic DNA of three dogs with the different genotypes are shown.

**Figure 4:**
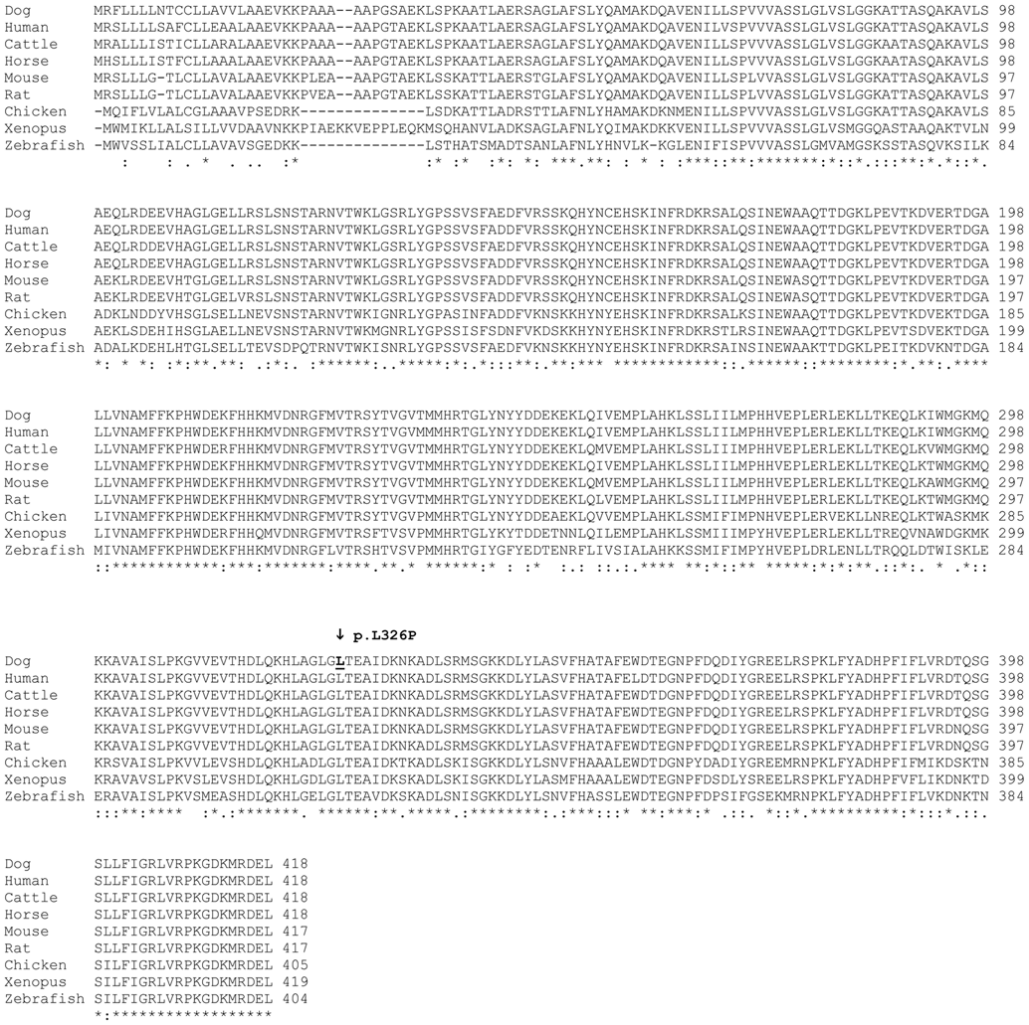
Multispecies alignment of the SERPINH1 protein sequence. The SERPINH1 protein sequence is highly conserved among all vertebrates. The p.L326P mutation in OI Dachshunds is indicated by an arrow. It affects a leucine residue, which is perfectly conserved from human to zebrafish across all investigated species. The sequences for the alignment were taken from the following accessions: XP_542305 (dog), NP_001226 (human), NP_001039528 (cattle), XP_001494735 (horse), NP_033955 (mouse), NP_058869 (rat), NP_990622 (chicken), NP_001080259 (Xenopus laevis), NP_571279 (zebrafish).

**Figure 5:**
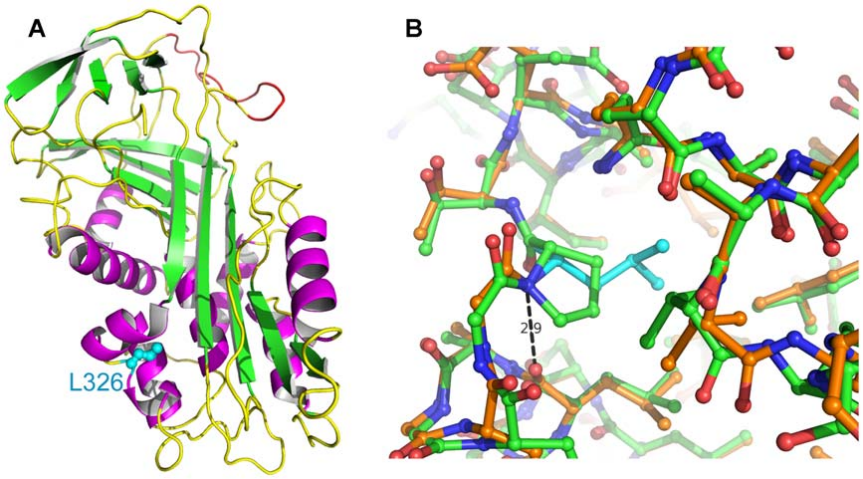
Homology models of wildtype and mutant SERPINH1. (A) Ribbon model of SERPINH1. Leu326 is located on the lower left with its side-chain drawn as ball-and-stick in cyan. The segment homologous to the reactive center loop of antiproteolytically active serpins is shown in red. Helices are colored magenta, sheets are depicted in green. (B) Close-up of the mutation site. Wildtype amino acids have carbon atoms colored in orange with the exception of Leu326 (cyan), while the mutant carbon atoms are drawn in green. Oxygens are shown in red and nitrogen atoms in blue. The H-bond between Leu326-NH and Leu321-O is indicated with a distance of 2.9 Å. Some distortions are visible around the mutation site.

As the quality of dog genome annotation is still far from perfect, we inferred the gene annotation of the mapped interval from the corresponding human interval. The dog OI interval corresponds to two human segments from 3.59–3.84 Mb and from 71.30–76.48 Mb on HSA 11. The two human intervals contain 98 annotated genes including 8 annotated pseudogenes (NCBI MapViewer, build 36.3). A careful inspection of these genes and database searches of their presumed function revealed *SERPINH1* as a functional candidate gene within the critical interval at 26.0 Mb on CFA 21. *SERPINH1* encodes a serine protease inhibitor, also called heat shock protein 47 (HSP47) or collagen binding protein 1. *Serpinh1* deficient mice die at around day 11 of development due to defective collagen synthesis [14] and it was shown that *Serpinh1^−/−^* fibroblasts produce abnormally thin and branched collagen type I fibres [15]. In order to further validate *SERPINH1* as positional candidate gene for OI, we genotyped two gene associated microsatellite markers derived from the surrounding genome sequence of CFA 21 (Table S1). The obtained LOD score of 4.1 conclusively confirmed the linkage of OI to the candidate gene region in the Dachshund families. All OI affected dogs showed homozygosity at both tested microsatellites and all genotyped parents had one copy of the disease associated haplotype. We therefore investigated whether mutations in the canine *SERPINH1* gene might be responsible for the OI phenotype. We designed PCR primers for the amplification of the four coding exons and determined the genomic sequence of two affected and two control dogs. This analysis revealed twelve polymorphisms including three non-synonymous substitutions (Table 1). Of these polymorphisms only a single SNP located in *SERPINH1* exon 5 (c.977T>C; Figure 3) showed perfect association to the OI phenotype (Table 2, Figure S1). All 11 affected dogs were homozygous C/C and all 13 known carriers were heterozygous C/T. One grandmother and 16 out of 22 healthy full- and half-sibs of OI affected dogs were also heterozygous C/T. None of 66 unrelated healthy Dachshunds had the homozygous C/C genotype, but twelve of them were also presumed carriers with the C/T genotype. Thus the allele frequency of the deleterious C-allele within the unrelated Dachshunds was 18%. The mutation was encountered in wire-haired and short-haired Dachshunds. The mutant C-allele was absent from 79 control dogs from 75 diverse dog breeds (Table S2). RT-PCR on bone cDNA confirmed that the *SERPINH1* RNA is normally spliced in an affected dog. We sequenced the cDNA from an affected dog and it did contain the mutant C-nucleotide at position +977 confirming that the mutant mRNA is expressed at normal levels. The c.977T>C substitution is predicted to result in an exchange of a highly conserved leucine to a proline in the SERPINH1 protein sequence (p.L326P, Figure 4). We modeled the wildtype and mutant SERPINH1 protein structures based on experimentally determined structures of serpins and found that the p.L326P mutation indeed affects the three-dimensional structure of SERPINH1 (Figure 5).

**Table 1:** *SERPINH1* gene polymorphisms

Polymorphism (cDNA)^a^	Position within *SERPINH1* gene	Protein	Association with OI^b^
c.-28C>T	exon 2, 5′-UTR	–	Homozygous mutant genotype in OI and control dogs
c.47T>C	exon 2	p.V16A	Mutant allele only in control dogs
c.83C>T	exon 2	p.A28V	Homozygous mutant genotype in OI and control dogs
c.471G>A	exon 2	silent	Homozygous mutant genotype in OI and control dogs
c.621G>A	exon 2	silent	Mutant allele only in control dogs
c.622+8T>C	intron 2	–	Mutant allele only in control dogs
c.721+52G>A	intron 3	–	Mutant allele only in control dogs
c.885A>G	exon 4	silent	Mutant allele also in a healthy dog from another breed
c.954+28T>C	intron 4	–	Mutant allele only in control dogs
**c.977T>C**	**exon 5**	**p.L326P**	**Homozygous mutant genotypes exclusively in OI Dachshunds**
c.1059C>T	exon 5	silent	Homozygous mutant genotype in OI and control dogs
c.1107C>T	exon 5	silent	Mutant allele only in control dogs

a Numbering refers to accession no. XM_542305 (this RefSeq model RNA is incorrectly annotated as *SERPINH2*, it does represent the putative canine *SERPINH1* mRNA sequence)

b The canine genome reference sequence was considered to represent the wildtype state. Sequence variants with respect to this sequence are designated “mutant” alleles or genotypes.

**Table 2:** *SERPINH1* genotype frequencies

		Dachshunds				Other breeds
*SERPINH1*		OI affected (n = 11)	OI carrier (n = 13)	Control, related (n = 23)	Control, unknown relationship (n = 66)	Controls (n = 79)
c.-28C>T	CC			4		
	CT		11	12		
	TT	11	2	7		
c.47T>C	TT	11	6	12		
(p.V16A)	TC		7	8		
	CC			3		
c.83C>T	CC			4		
(p.A28V)	CT		11	12		
	TT	11	2	7		
c.471G>A	GG			4		
	GA		11	12		
	AA	11	2	7		
c.621G>A	GG	11	12	19		
	GA		1	4		
	AA					
c.622+8T>C	TT	11	12	19		
	TC		1	4		
	CC					
c.721+52G>A	GG	10^a^	11	14		
	GA		2	9		
	AA					
c.885A>G	AA			3		
	AG		10	10		
	GG	10^a^	3	10		
c.954+28T>C	TT	10^a^	12	19		
	TC		1	1		
	CC			3		
**c.977T>C**	**TT**			**6**	**54**	**79**
**(p.L326P)**	**TC**		**13**	**17**	**12**	
	**CC**	**11**				
c.1059C>T	CC			4		
	CT		11	12		
	TT	11	2	7		
c.1107C>T	CC	11	12	19		
	CT		1	4		
	TT					

a Low DNA quality prevented the determination of the genotypes in one amplicon from a single affected dog.

## Discussion

We have applied an efficient SNP-based homozygosity mapping strategy to map the causative gene for OI in Dachshunds using only five affected dogs and five obligate carriers. The special population structure of purebred dog breeds with a limited amount of inbreeding on the one hand increases the occurrence of recessive phenotypes and on the other hand provides ideal prerequisites to map the underlying genes for these traits [16]. In this study we did not have enough high-quality DNA samples of unrelated Dachshunds for a genome-wide association study. However, the homozygosity mapping approach for this recessive trait basically required only samples from the five affected dogs to map the causative gene to one unique chromosome segment of 5.82 Mb. Adding the five obligate carriers further reduced this interval to 4.74 Mb. Thus the use of genome-wide canine SNP genotyping data enables very efficient positional cloning projects of Mendelian traits even if only very few samples are available.

The mapped OI interval contains a very good functional candidate gene, *SERPINH1*. We found a non-synonymous mutation in this gene, which is perfectly associated with the OI phenotype in Dachshunds, and confirmed the presence of this mutation on the genomic DNA and mRNA level. Although we cannot provide functional proof of the causality of the mutation at this time, the wealth of functional data, which are available for the *SERPINH1* gene, strongly supports the hypothesis that p.L326P is indeed the causative mutation. SERPINH1 or HSP47 is a molecular chaperone of the serpin family. It promotes the correct folding of the collagen I triple helix [17]. This triple-helical structure would normally not be stable at temperatures above 35 °C. SERPINH1 is present in high concentration in the endoplasmic reticulum and specifically binds to and stabilizes the triple helices of nascent collagens [18]–[20]. Apparently the complete absence of SERPINH1 leads to embryonic lethality due to deficiencies in several types of collagen [14]. It is an evolutionary conserved protein with 97% identity between human and dog and 64% identity between human and zebrafish. The p.L326P mutation lies within the conserved serpin domain and the wildtype leucine is conserved across all SERPINH1 sequences while in other, more distantly related serpins like antitrypsin or ovalbumin, it is conservatively replaced by isoleucine, valine or methionine. It is located at the interface of helices hB, hC and hI (Figure 5A) [21]. Leu326 has backbone dihedral angles Φ/Ψ of about −90/+88 degrees. Proline has a Φ-angle restricted to about −60 degrees due to its five-membered ring and most frequently Ψ-angles of −45 or +135 degrees. Therefore, it is likely that the mutation L326P results in an increased strain. Furthermore, in the wildtype Leu326 donates a main-chain H-bond to Leu321 that is not possible with the imino acid proline (Figure 5B). Thus it is conceivable that this mutation affects the proper folding and stability of the native conformation, possibly reducing the protein level significantly. Additionally, this non-conservative amino acid substitution could affect the ability of SERPINH1 to bind and stabilize collagen triple helices. We speculate that the p.L326P mutation in OI affected dogs probably does not represent a complete null allele but has some residual activity, which results in live-born dogs with a severe form of OI instead of the embryonic lethality seen in *Serpinh1* knock-out mice. The phenotype of OI affected dogs primarily indicates a deficiency in collagen I, the most abundant collagen, whereas basal membranes, which contain collagen IV, do not seem to be severely altered [13].

Our finding of a *SERPINH1* p.L326P mutation in dogs with OI provides a valuable model for human medicine and identifies *SERPINH1* as a fifth OI gene in addition to *COL1A1*, *COL1A2*, *CRTAP*, and *LEPRE1*. It has already been shown that a functional SNP in the promoter of the human *SERPINH1* gene is associated in African American women with an increased risk for preterm premature rupture of membranes [22]. Our study indicates that coding mutations of the *SERPINH1* gene might be responsible for recessive forms of human OI, where no mutation in the four known OI genes has been found. It has been proposed to develop SERPINH1 binding molecules as drugs against fibrosis [23]. The findings of our study emphasize that such a therapeutic strategy will have to be very carefully adjusted in order not to have adverse effects on the physiological production of collagen.

In conclusion, we have identified the p.L326P mutation in the canine *SERPINH1* gene as the candidate causative mutation for OI in Dachshunds. This result allows genetic testing and eradication of a lethal disease from the Dachshund breeding population. Our study also provides a defined animal model and a novel genetic mechanism for a lethal or severely debilitating human hereditary disease.

## Materials and Methods

### Animals

We collected samples from OI affected rough-coated Dachshunds (n = 11), their healthy littermates (n = 22), sires (n = 4), dams (n = 7), and one grandmother. We performed parentage verification to confirm the pedigree documentation from the breeders (Figure S1). In addition, we collected two Dachshunds recorded as sires of OI affected puppies. Parents of affected offspring were classified as obligate carriers (n = 13). We also collected 66 unrelated healthy Dachshunds resulting in a total of 113 samples from the Dachshund breed. Furthermore, we sampled 79 control dogs from 75 different breeds for the re-sequencing of *SERPINH1* exon 5 (Table S3).

### DNA and RNA extraction

Genomic DNA was isolated from blood or tissue using the Nucleon Bacc2 kit (GE Healthcare). Total RNA was isolated from bone or skin using Trizol reagent according to the manufacturer's instructions (Invitrogen).

### Linkage analysis in candidate genes

Microsatellite markers were amplified using the Multiplex PCR Kit (Qiagen) and fragment size analyses were determined on an ABI 3730 capillary sequencer (Applied Biosystems) and analyzed with the GeneMapper 4.0 software (Applied Biosystems). Twopoint parametric linkage analysis under the assumption of OI segregating as a biallelic autosomal recessive trait with complete penetrance was performed with Merlin software version 1.1.2 [24]. The frequency of the mutant allele in the considered population was unknown and there were no data available that would have made it possible to estimate the frequency in a reliable manner. For the calculations a frequency of 0.001 for the mutant allele was assumed. The LOD score test statistic was used to estimate the proportion of linked families and the corresponding maximum heterogeneity LOD score. Within the available families, a maximum LOD score of 5.573 would have been possible. To reconstruct the most likely haplotypes, we applied the ‘best’ option of the Merlin software.

### Mapping of the OI mutation

Genomic DNA from five affected Dachshunds and five carriers was genotyped on the canine Affymetrix version 2 SNP genotyping microarray (49,663 SNPs). The results were analyzed with PLINK [http://pngu.mgh.harvard.edu/~purcell/plink/]. To identify extended homozygous regions with allele sharing across all five affected animals the options –homozyg-group and –homozyg-match were applied. All given positions correspond to the build 2.1 dog genome assembly [http://www.ncbi.nlm.nih.gov/projects/mapview/map_search.cgi?taxid=9615].

### Mutation analysis

Primers for the amplification of each of the four *SERPINH1* coding exons with flanking regions were designed with the software Primer3 [http://frodo.wi.mit.edu/cgi-bin/primer3/primer3_www.cgi] after masking repetitive sequences with RepeatMasker (Smit, A.F.A and Green, P. [http://repeatmasker.genome.washington.edu/]). The sequences of the primers are listed in Table S4. For the mutation analysis PCR products were amplified of two affected and two unrelated healthy dogs using TopTaq polymerase (Qiagen). The subsequent re-sequencing of the PCR products was performed after rAPid alkaline phosphatase (Roche) and exonuclease I (New England Biolabs) treatment using both PCR primers with the ABI BigDye Terminator Sequencing Kit 3.1 (Applied Biosystems) on an ABI 3730. Sequence data were analyzed with Sequencher 4.8 (GeneCodes).

### RT–PCR

Aliquots of 1 µg total RNA were reverse transcribed into cDNA using 20 pmol (T)_24_V primer and Omniscript reverse transcriptase (Qiagen). Two microliters of the cDNA were used as a template in PCR. PCR reactions were performed as described above and primer sequences are given in Table S5. The canine *SERPINH1* cDNA sequence was deposited under accession FN395288 in the EMBL nucleotide database.

### Homology modeling

Models of wildtype and mutant SERPINH1were produced employing 3D-JIGSAW [25],[26] with template PDB entries1OO8 and 1OPH [27]. Figures were prepared using PyMOL [http://www.pymol.org].

## Supporting Information

Figure S1 Pedigrees of sampled Dachshunds. Animals genotyped on the SNP chip are indicated by asterisks. The genotypes for the *SERPINH1* c.977T>C mutation are given below the symbols.
(0.02 MB PDF)

Table S1 Microsatellites.
(0.02 MB XLS)

Table S2 Results of homozygosity mapping.
(0.04 MB XLS)

Table S3 List of dog breeds tested for *SERPINH1* exon 5 SNPs.
(0.02 MB XLS)

Table S4 Primer sequences for the amplification of canine *SERPINH1* coding exons.
(0.02 MB XLS)

Table S5 Primer sequences for the amplification of canine *SERPINH1* cDNA.
(0.03 MB XLS)

Video S1 Affected Dachshund puppy showing wobbly gait due to ligament hyperlaxity and unstable joints, especially the carpal joints.
(6.96 MB AVI)
