# Supplementary material for: A Missense Mutation in the SERPINH1 Gene in Dachshunds with Osteogenesis Imperfecta
Source: PLoS Genet. 2009 Jul 24;5(7):e1000579. doi: 10.1371/journal.pgen.1000579 (PMC2708911; doi:10.1371/journal.pgen.1000579)

Figure S1. Pedigrees of sampled Dachshunds. Animals genotyped on the SNP chip are indicated by asterisks. The genotypes for the *SERPINH1* c.977T>C mutation are given below the symbols.

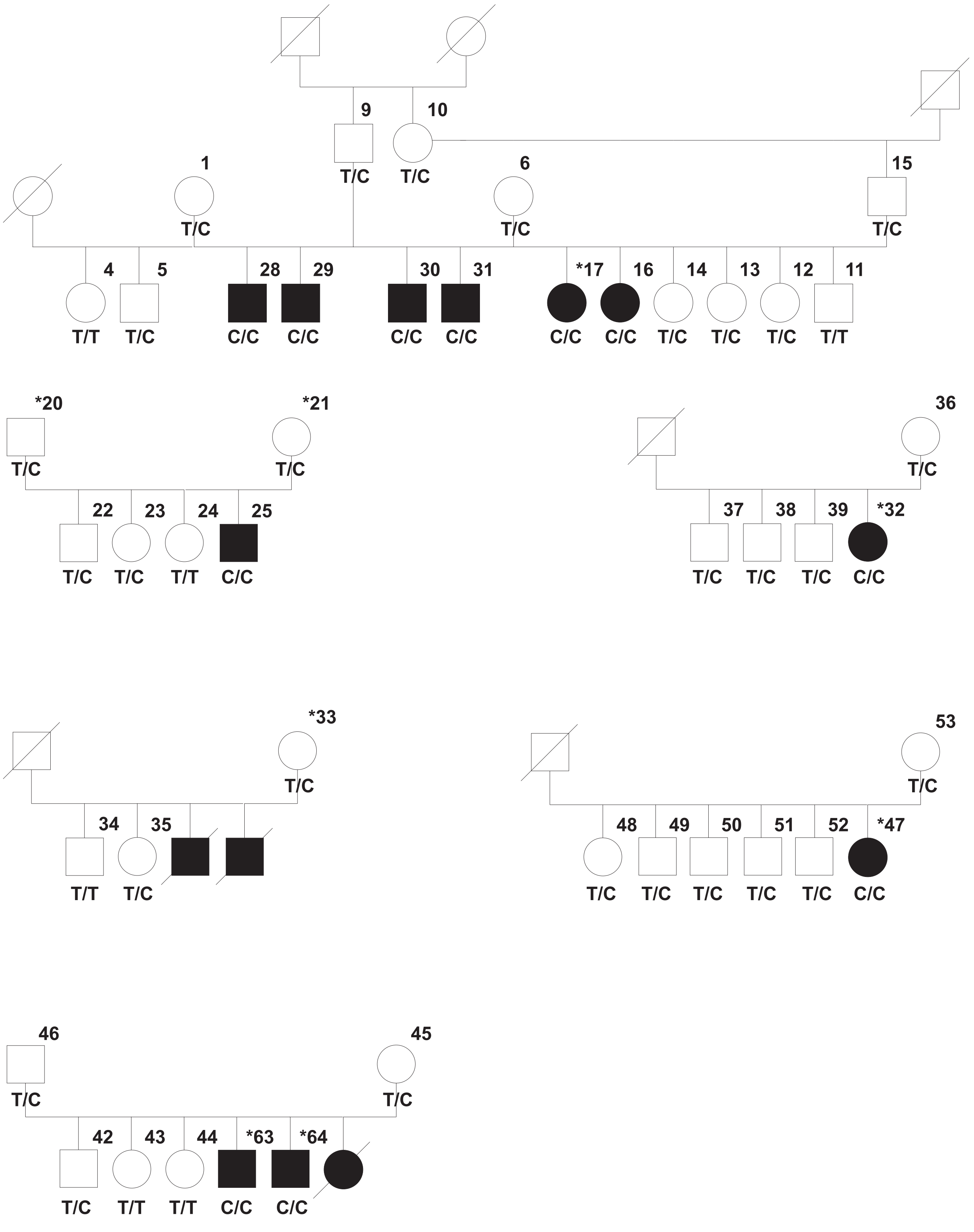

Supplement: Figure S1 — Pedigrees of sampled Dachshunds. Animals genotyped on the SNP chip are indicated by asterisks. The genotypes for the SERPINH1 c.977T>C mutation are given below the symbols. (0.02 MB PDF) [file pgen.1000579.s001.pdf]
